# Association of advanced glycation end products in Dupuytren disease

**DOI:** 10.1186/s13018-018-0848-4

**Published:** 2018-06-07

**Authors:** Fumiaki Takase, Yutaka Mifune, Atsuyuki Inui, Yasuhiro Ueda, Takeshi Kataoka, Takeshi Kokubu, Ryosuke Kuroda

**Affiliations:** Department of Orthopedic Surgery, Kobe University Graduate School of Medicine, 7-5-1, Kusunoki-cho, Chuo-ku, Kobe, 650-0017 Japan

**Keywords:** Dupuytren’s disease, Advanced glycation end products (AGEs), Oxidation

## Abstract

**Background:**

Advanced glycation end products are associated with aging, hyperglycemia, and oxidative stress. Accumulation of advanced glycation end products can cause various pathological conditions; however, the association of Dupuytren’s disease with advanced glycation end products has not been demonstrated yet. The aim of this study is to investigate the association of Dupuytren’s disease with advanced glycation end products.

**Methods:**

Normal palmar fascia from five patients with carpal tunnel syndrome (control group) and Dupuytren’s cords from five patients (Dupuytren’s disease group) were harvested. The tissues were stained using an anti-advanced glycation end products antibody, anti-receptor for advanced glycation end products antibody, and an anti-reactive oxygen species modulator 1 antibody. The expression of nicotinamide adenine dinucleotide phosphate oxidase-1 and nicotinamide adenine dinucleotide phosphate oxidase-4 genes was also assessed using real-time PCR. For in vitro analysis, the cells harvested from the control and Dupuytren’s disease groups were used. After 3 days of exposure to four types of media (control group, control + advanced glycation end products group, Dupuytren’s disease group, Dupuytren’s disease + advanced glycation end products group), superoxide detection reagent was detected using a total reactive oxygen species/superoxide detection kit.

**Results:**

Immunostaining of the palmar fasciae of the Dupuytren’s disease group showed higher expressions of advanced glycation end products and receptor for advanced glycation end products than that in the control group. The expression of nicotinamide adenine dinucleotide phosphate oxidase oxidase-1 and nicotinamide adenine dinucleotide phosphate oxidase-4 as well as reactive oxygen species modulator 1, an oxidatively damaged protein, was also higher in the Dupuytren’s disease group than in the control group. In an in vitro cell culture, the addition of advanced glycation end products to the Dupuytren’s disease-derived cells produced more superoxide free radicals.

**Conclusions:**

These data suggest that the advanced glycation end products receptor for advanced glycation end products interaction produced free radicals via nicotinamide adenine dinucleotide phosphate oxidase activation in Dupuytren’s disease patients. Further studies are required to confirm these results.

## Background

Dupuytren’s disease (DD) is a benign progressive disease of the palmar fascia that leads to flexion contracture of the fingers. Histologically, hyper collagen synthesis by fibroblasts and myofibroblasts has been thought to have a central role in the disease. The etiology of DD has been controversial since the disease was first described in 1832 [1]. Ling reported that genetic factors are important in the pathogenesis of DD [2]. Additionally, environmental factors, such as heavy manual work, smoking, and alcohol, are known to be associated with DD [3, 4]. Moreover, Geoghegan et al. reported that diabetes mellitus was a significant risk factor for DD [5].

Recently, advanced glycation end products (AGEs) have been gaining attention because deposition of AGEs in organs and tissues can cause various diseases, such as arteriosclerosis [6], cataract [7], renal failure [8], and osteoporosis [9]. AGEs are formed by the Maillard reaction, a nonenzymatic reaction between the ketone or aldehyde groups of sugars and the amino groups of proteins [10], and are known to increase oxidative stress and inflammation through binding to the receptor for AGEs (RAGE) [11]. Accumulation of AGEs is also accelerated by hyperglycemia leading to the development of diabetic complications [12]. Although both DD and AGEs are associated with diabetes mellitus, the association of DD with AGEs has not been demonstrated yet. In this study, we assessed the deposition of AGEs and oxidative stress markers in the palmar fasciae of patients with DD.

## Methods

### Patients and preparation

The protocol of this study was approved by the institutional review board of Kobe University Hospital. Normal palmar fasciae from five patients with carpal tunnel syndrome (CTS) (control group) and Dupuytren’s morbid cords from five patients without diabetes mellitus (DD group) were harvested during the surgical treatment. We confirmed that both CTS and DD patients did not suffer from diabetes through a pre-operative blood examination. The tissues were frozen quickly at − 80 °C, and a part of the tissues was prepared for biochemical measurements. The remaining tissues were cut into 10-μm-thick sections for staining. The slides were dried at room temperature and fixed in 4% paraformaldehyde.

For in vitro analysis, part of the excised tissue samples was also cut into small pieces (1–2 mm) and plated onto dishes with Dulbecco’s Modified Eagle’s Medium (Sigma, St. Louis, MO, USA) supplemented with 10% fetal bovine serum) (Sigma, St. Louis, MO, USA) and 1% penicillin–streptomycin (Sigma, St. Louis, MO, USA) (regular medium) under sterile conditions. The dishes were maintained in a 5% CO2 chamber at 37 °C. After adhesion of cells to the dishes was observed, the cells were washed in phosphate-buffered saline, detached by using 0.05% trypsin–0.02% EDTA (Wako, Osaka, Japan), and pelleted in 75-cm2 cell culture flasks with regular medium. The cells were then seeded onto 12-well plates at a concentration of 5.0 × 104 cells per well. After the cells had reached 70% confluence, they were cultured in four groups: palmar fascia-derived cells from carpal tunnel syndrome (CTS) patients with regular medium only (control group), palmar fascia-derived cells from CTS patients with regular medium plus AGEs (200 μg/ml, Trans Genic, Kobe, Japan) (control + AGEs group), palmar fascia-derived cells from DD patients with regular medium only (DD group), and palmar fascia-derived cells from DD patients with regular medium plus AGEs (DD + AGEs group). All experiments were performed with cells from the second or third passage, and the same passage of cells was used for each experiment.

### Hematoxylin-eosin (HE) staining and immunohistochemistry

HE staining was performed to observe the histological differences between the control group and DD group. The sections were incubated with alpha-smooth muscle actin (alpha-SMA) antibody (Sigma-Aldrich, St Louis, MO) to confirm the expression of myofibroblasts that characterize DD. The sections were also incubated at 4 °C overnight with the following primary antibodies: anti-AGEs antibody (10 μg/ml; Abcam, Cambridge, UK), anti-RAGE antibody (10 μg/ml; Abcam), and anti-reactive oxygen species modulator 1 (ROMO1) antibody (0.7 μg/ml; Abcam). After incubation with the primary antibodies, the sections were incubated with the secondary antibody, Alexa Fluor 594 (20 μg/ml; Abcam), and counterstained with 4′,6-diamidino-2-phenylindole (DAPI). Digital images of AGEs, RAGE, ROMO1 (red), and nuclear (blue) staining were captured by using a Bio Zero BZ-8000 (Keyence, Osaka, Japan). The positive areas were quantified by using selective coloring in Adobe Photoshop (CS6; Adobe Systems, San Jose, CA, USA) [13].

### Expression of superoxide in the cells

After 3 days of exposure to four types of media (control group, control + AGEs group, DD group, DD + AGEs group), superoxide detection reagent was detected by using a Total ROS/Superoxide detection kit (Enzo Life Science, Plymouth Meeting, PA, USA), and the cells were counterstained with DAPI. Digital images were captured from five non-overlapping microscopic fields per well by using the Bio Zero BZ-8000 (Keyence, Osaka, Japan), and superoxide-positive cells were counted.

### Real-time polymerase chain reaction (PCR)

Total RNA was extracted from the cells and the tissues by using an RNeasy Mini Kit (Qiagen, Valencia, CA, USA). Total RNA was reverse transcribed into single-strand cDNA by using a High-Capacity cDNA Reverse Transcription Kit (Applied Biosystems, Foster City, CA, USA). A real-time PCR was performed in triplicate on the cDNA with an Applied Biosystems 7900HT Fast Real-time PCR System and SYBR Green reagents (Applied Biosystems, Foster City, CA, USA). The results were normalized to housekeeping gene expression levels and expressed relative to the control (untreated) culture levels by using the 2^-∆∆Ct^ method. We used the primers for RAGE, nicotinamide adenine dinucleotide phosphate oxidase (Nox)-1, and Nox-4.

The following primer sequences were used: GAPDH: F 5′-CCA CCC ATG GCA AAT TCC-3′ R 5′-GAT GGG ATT TCC ATT GAT GAC A-3′; RAGE: F 5′-AGG AGC GTG CAG AAC TGA AT-3′ R 5′-TTG GCA AGG TGG GGT TAT AC-3′; Nox1: F 5′-GTA CAA ATT CCA GTG TGC AGA CCA C-3′ R 5′-CAG ACT GGA ATA TCG GTG ACA GCA-3′; Nox4: F 5′-CTC AGC GGA ATC AAT CAG CTG TG-3′ R 5′-AGA GGA ACA CGA CAA TCA GCC TTA G-3′.

### Statistical analysis

Data are presented as means ± standard deviations of independent experiments. These analyses were performed by using EZR (Saitama Medical Center, Jichi Medical University, Saitama, Japan), which is a graphical user interface for R (The R Foundation for Statistical Computing, Vienna, Austria) [14]. For comparisons of two groups, Welch’s *t* test was performed. A value of *p* < 0.05 was considered as indicating statistical significance.

## Results

### In vivo experiments

#### HE staining and immunohistochemistry

**Fig. 1:**
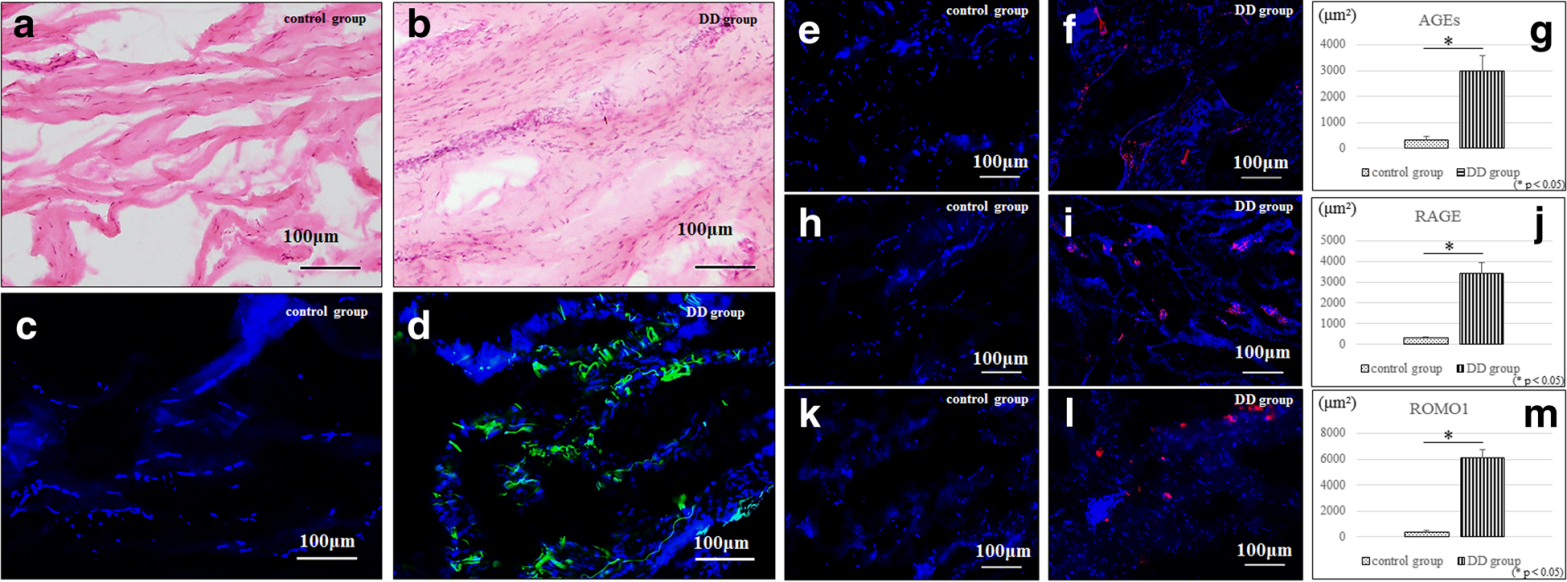
HE staining and immunohistochemistry (alpha-SMA, AGEs, RAGE, ROMO1). There were more collagen fibers and nuclei in the DD group than in the control group (**a**, **b**). No expression of alpha-SMA was observed in the control group, whereas strong expressions were observed in the DD group (**c**, **d**). Increased expressions of AGEs were seen in the palmar fascia of the DD group than in the control group (**e**, **f**, **g**). Regarding RAGE and ROMO1, there was a little expression in the control group (**h**, **k**), but their expression in the DD group was significantly higher (**i**, **j**, **l**, **m**).

Greater collagen fiber formation with cell aggregation was observed in the DD group than in the control group (Fig. 1a, b). No expression of alpha-SMA was observed in the control group, whereas strong expression was observed in the DD group (Fig. 1c, d). More AGE expressions were seen in the palmar fasciae of the DD group than in that of the control group (Fig. 1e–g). There was also little expression of RAGE and ROMO1 in the control group (Fig. 1h, k). The expression of RAGE and ROMO1 was significantly higher in the DD group (Fig. 1i, j, l, and m).

#### Real-time PCR

**Fig. 2:**
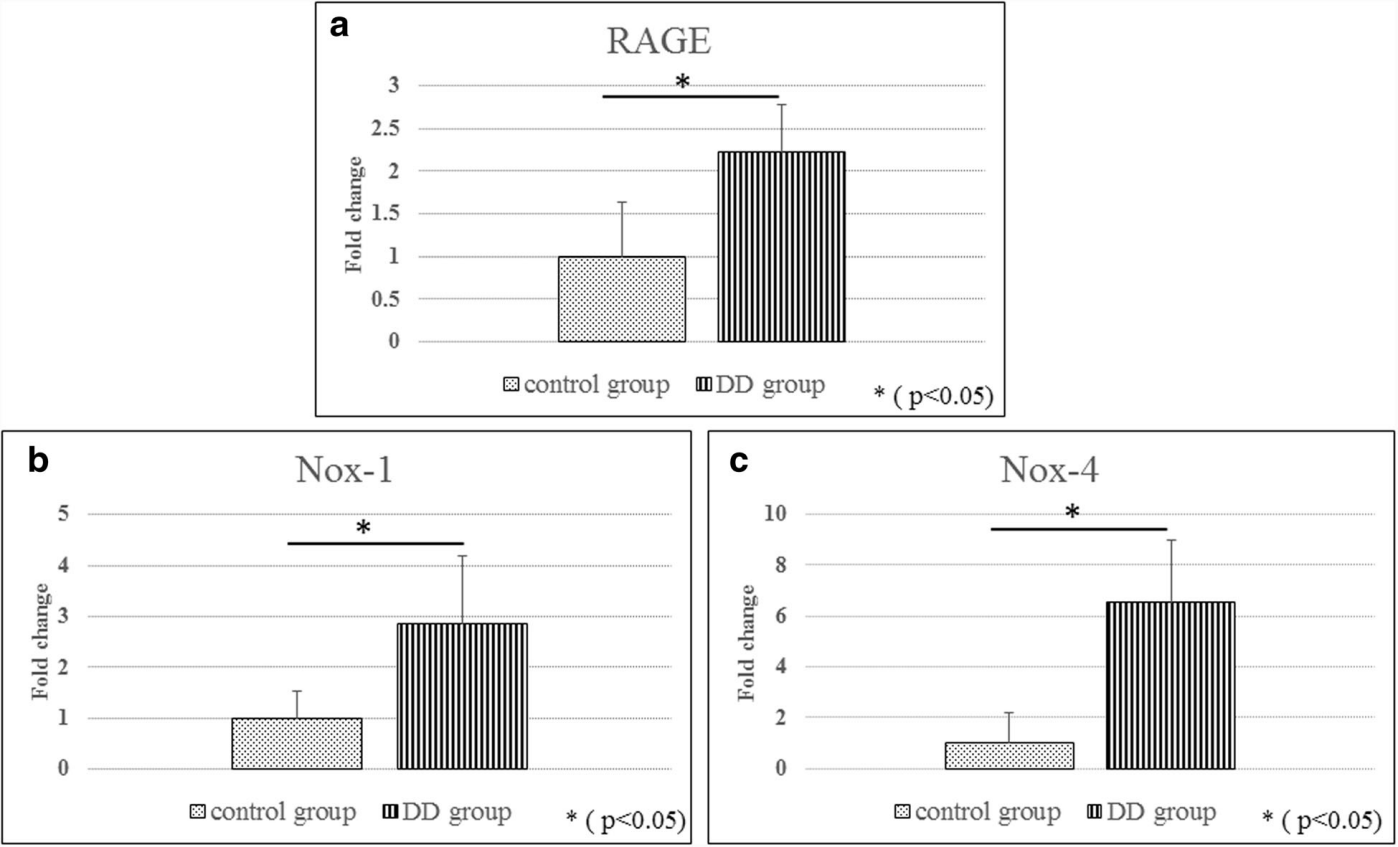
Real-time PCR. Values were normalized to GAPDH expression. RAGE gene expression was significantly higher in the DD group than in the control group (**a**). Nox-1 and Nox-4 gene expressions were also significantly higher in the DD group than in the control group (**b**, **c**)

RAGE, Nox-1, and Nox-4 gene expressions were significantly higher in the DD group than in the control group (Fig. 2a, b, and c, respectively).

### In vitro experiments

#### Expression of superoxide in the cells

**Fig. 3:**
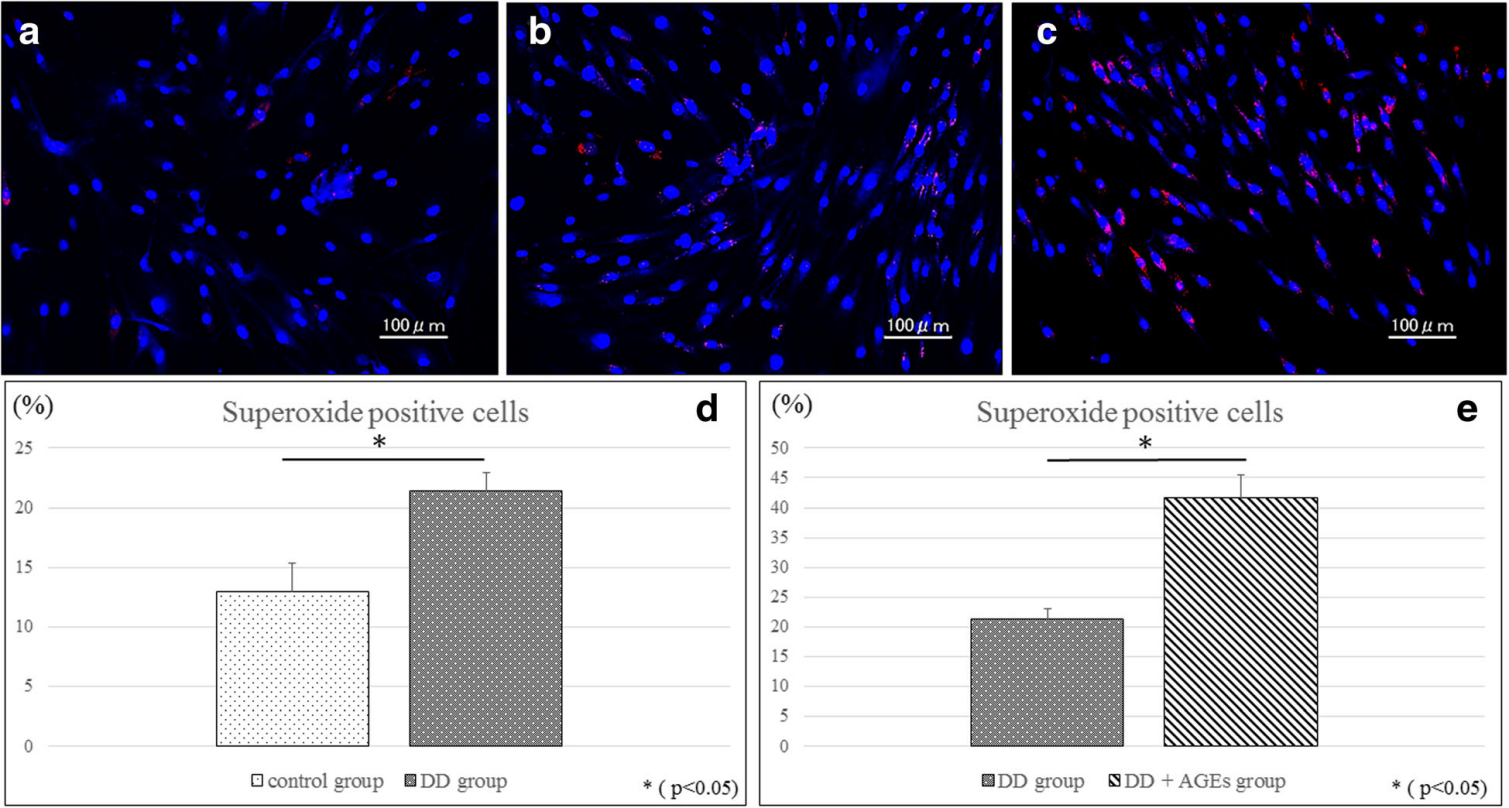
Expression of superoxide in the cells. Only a few superoxide-positive cells were observed in the control group (**a**). There were significantly more superoxide-positive cells in the DD group than in the control group (**b**, **d**). There were also significantly more superoxide-positive cells in the DD + AGEs group than in the DD group (C, E)

Only a few superoxide-positive cells were observed in the control group (Fig. 3a), whereas there were significantly higher numbers of superoxide-positive cells in the DD group (Fig. 3b, d). The addition of AGEs caused more superoxide formation in DD-derived cells (Fig. 3c, e).

#### Real-time PCR

**Fig. 4:**
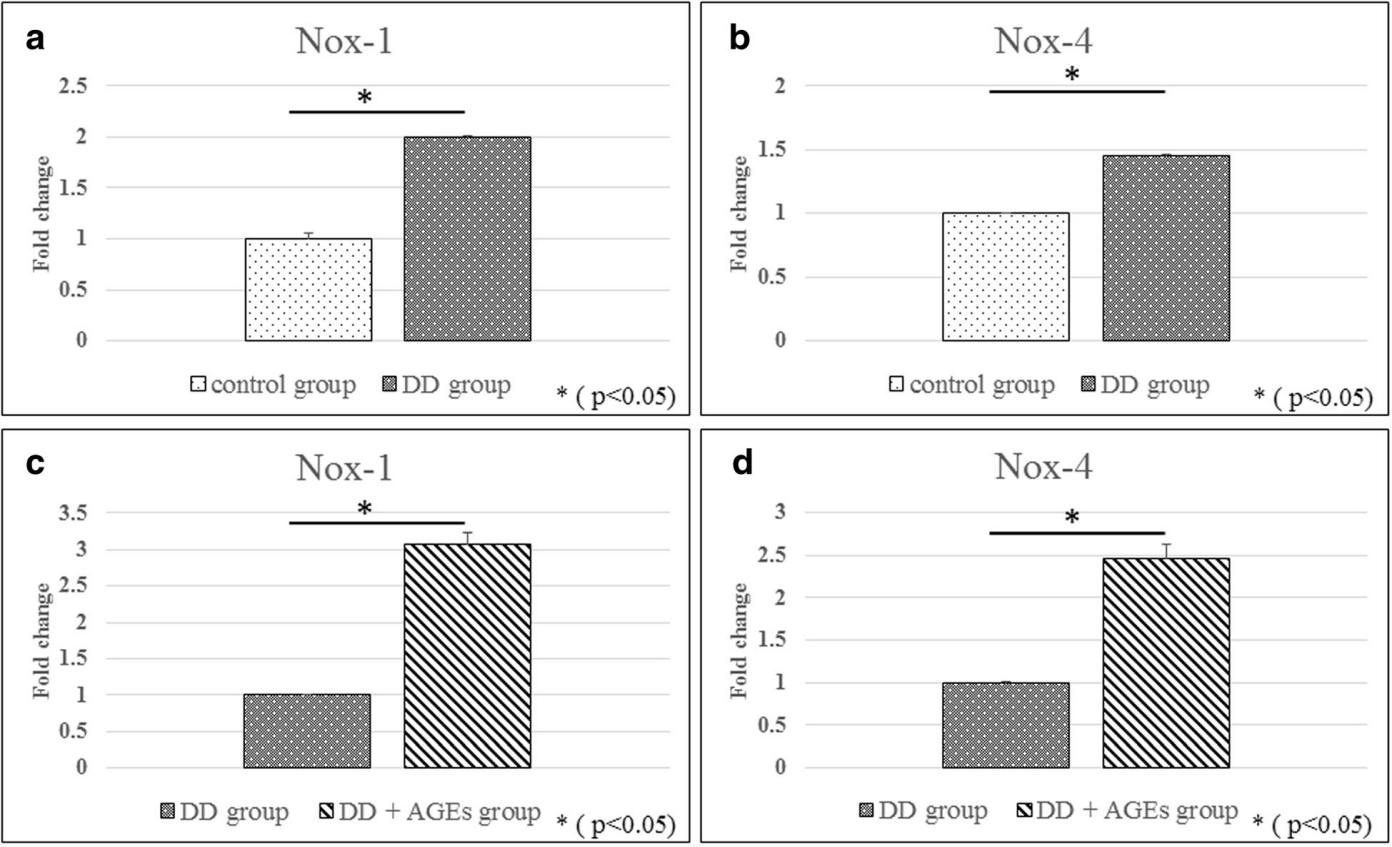
Real-time PCR. Values were normalized to GAPDH expression. Nox-1 and Nox-4 gene expressions were significantly higher in the DD group than in the control group (**a**, **b**). Nox-1 and Nox-4 gene expressions were significantly higher in the DD + AGEs group than in the DD group (**c**, **d**)

Nox-1 and Nox-4 gene expressions were significantly higher in the DD group than in the control group (Fig. 4a, b). The addition of AGEs into the medium significantly increased Nox-1 and Nox-4 gene expressions relative to those in the DD group (Fig. 4c, d).

## Discussion

Although numerous studies have been conducted on this topic, the pathogenesis of DD remains unclear. A major biochemical abnormality in DD tissue is an increase of fibroblasts with collagen synthesis. The ratio of type III to type I collagen has been found to be higher in DD than in normal tendons [15, 16]. Excessive expression of myofibroblasts is also associated with excessive collagen production [17]. In our study, an increase in cell numbers with dense collagen fibers as well as myofibroblasts that expressed alpha-SMA was seen in the palmar fasciae of patients with DD, which is consistent with the results of the previous report mentioned above.

Regarding oxidative stress, microangiopathic ischemia causes production of free radicals that stimulate cytokine release and fibroblast proliferation [18]. Local ischemia causes ATP to be broken down into hypoxanthine and xanthine dehydrogenase that are then converted into xanthine oxidase. Then, enzymatic conversion of the produced hypoxanthine to xanthine by xanthine oxidase generates free radicals. Excess free radicals are thought to contribute to the progression of atherosclerosis by damaging blood vessel walls and cause further local ischemia [19]. Free radicals can attack collagen directly, and free-radical-mediated crosslinking of collagen increases tissue stiffness [20]. Murrell et al. reported that production of free radicals might be an important factor in the pathogenesis of DD [21]. Their results showed that the concentration of hypoxanthine in the palmar fascia was higher in patients with DD than in controls. They also reported that altered concentrations of free radicals stimulated fibroblast proliferation and concluded that the results might help explain fibroblast proliferation in DD [22].

Collagen is the main component of extracellular matrix (ECM) in the body, and its glycation process can affect intermolecular and intramolecular crosslinks of collagen. The crosslink reactions lead to structural alterations of collagen, such as increased stiffness and resistance to proteolytic digestion. AGEs are formed by nonenzymatic reaction of glucose with proteins. Clinically, accumulation of AGEs in connective tissues has been found to be associated with increased tendon thickness and decreased shoulder joint mobility and upper extremity function [23]. Reportedly, AGEs crosslinking on type I collagen increased the stiffness of blood vessels [24, 25]. Balaba et al. reported that impairments in collagen metabolism were observed in patients with DD [26].

The effects of AGEs are classified as receptor independent or receptor dependent. The receptor-dependent effects of AGEs are caused by binding to various receptors, such as RAGE. RAGE is a transmembrane receptor of the immunoglobulin superfamily and was first characterized in 1992 [27]. The interaction between AGEs and RAGE promotes Nox activity, which results in activation of nuclear factor kappa B, followed by expression of inducible nitric oxide synthase and increased formation of free radicals [28]. Previously, seven Nox isoforms have been identified, and the Nox family is an important enzymatic source of free radicals. In the cardiovascular system, among the seven isoforms, the major source of free radicals was Nox-1 and Nox-4 because they were the main isoforms in vascular smooth muscle cells [29]. Koike et al. reported that AGEs significantly increased expression of Nox-1and Nox-4 mRNA in rat vascular smooth muscle cells [30].

The association of AGEs or Nox family in DD is not well known. Rosenbloom et al. reported that AGEs increased tissue stiffness in DD patients, but its mechanism was not fully described [31]. In our study, the addition of AGEs to DD-derived cells led to greater superoxide free radical formation. Increased AGE deposition and increased RAGE expression were also observed in the palmar fasciae of DD patients relative to those in the controls. The expressions of Nox-1 and Nox-4 as well as of ROMO1, an oxidatively damaged protein, were also higher in DD patients than in controls. These data suggested that the AGEs–RAGE interaction might produce free radicals via Nox activation in DD patients, and the pathogenesis of DD is associated with deposition of AGEs within the palmar fascia. Increased oxidative stress might induce increased AGE formation because glycation and oxidation influence each other [32].

This study had several limitations. First, we did not assess other AGE pathways besides of the receptor-dependent mechanism. Regarding the receptor-independent mechanism, ECM proteins are susceptible to AGE modification because of their slow turnover rate [12]. The expressions of type III, IV, and VI collagen were increased through upregulation of key profibrotic cytokines, such as TGF-β [33, 34] and connective tissue growth factor [35], following AGE modification of the ECM proteins. Second, the palmar fasciae from CTS patients were used as a control. Although there have been no reports about the morbid characteristics of the palmar fasciae in CTS patients, these controls were not healthy volunteers. Third, the sample size was small, and further studies with larger sample are required to confirm these results.

## Conclusions

Our results suggest that increased AGEs–RAGE interaction in DD patients generate more free radicals via Nox activation in these patients than in CTS patients. Therefore, it is possible that acceleration of the glycation and oxidation process contributes to the pathogenesis of DD. Further studies are required to confirm these results.

## Abbreviations

AGEs: Advanced glycation end products
CTS: Carpal tunnel syndrome
DAPI: 4′,6-diamidino-2-phenylindole
DD: Dupuytren’s disease
ECM: Extracellular matrix
HE: Hematoxylin-eosin
Nox: Nicotinamide adenine dinucleotide phosphate oxidase
PCR: Polymerase chain reaction
RAGE: Receptor for advanced glycation end products
ROMO: Reactive oxygen species modulator
SMA: Smooth muscle actin
